# Minimizing the Diagnostic Delay in Amyotrophic Lateral Sclerosis: The Role of Nonneurologist Practitioners

**DOI:** 10.1155/2020/1473981

**Published:** 2020-05-11

**Authors:** Martin Matharan, Stéphane Mathis, Sarah Bonabaud, Louis Carla, Antoine Soulages, Gwendal Le Masson

**Affiliations:** ^1^Department of Neurology, Nerve-Muscle Unit, CHU Bordeaux, Pellegrin Hospital, F-33096 Bordeaux, France; ^2^ALS Center, CHU Bordeaux, Pellegrin Hospital, F-33096 Bordeaux, France; ^3^University of Bordeaux, Neurocentre Magendie, Physiopathologie de la Plasticité Neuronale, F-33000 Bordeaux, France; ^4^INSERM, Neurocentre Magendie, Physiopathologie de la Plasticité Neuronale, F-33000 Bordeaux, France

## Abstract

**Introduction:**

Amyotrophic lateral sclerosis (ALS), usually fatal in a few years, is a neurodegenerative disorder where the diagnostic delay, although variable according to the studies, remains too long. The main objective of this study was to determine the average time to diagnose ALS and the role of each physician, general practitioner (GP), or specialist (neurologist or not) involved in the management of these patients. The secondary objective was to propose some simple schemes to quickly identify an ALS suspicion with the aim to reduce this delay. *Patients and Methods*. This retrospective study evaluated the diagnostic delay (and other intermediate delays) of 90 ALS patients registered in the ALS Center of Bordeaux (France) in 2013. The main clinical signs encountered (and their order of appearance) were studied.

**Results:**

The average diagnostic delay was 17 months, with a median diagnostic delay of 12 months. The average diagnostic delay was 2.7 months between the first symptoms and the first complaint to GP, followed by an additional 6.5 month delay before the patient's first visit to a neurologist. This period could be shortened, especially if GP performed additional tests quickly (*p*=0.01), as the time spent consulting various specialists often extends this crucial step. Overall, diagnostic delay accounted for 40% of the total duration of the disease progression.

**Conclusion:**

In relation to total survival time, the diagnostic delay of ALS appears to be proportionately very long, sometimes longer than that observed in previous studies (because it also included the total delay to diagnostic or treatment initiation). The rapid execution of useful additional tests by the first medical doctor, often GP (with the help of a neurologist), considerably reduces the diagnostic delay. The central role of GP seems to be crucial in the management of patients with ALS. The main objective is, of course, to initiate appropriate treatment and care as soon as possible. Finally, based on our results, we also provide a short practical diagram to help nonneurologist practitioners to quickly discuss the diagnosis of ALS in case of some specific symptoms (“red flags”).

## 1. Introduction

Amyotrophic lateral sclerosis (ALS) is a severe neurodegenerative disorder leading to death in 3–5 years [1]. As described by Jean-Martin Charcot himself [2], ALS affects both upper (UMN) and lower (LMN) motor neurons. Due to the known association with nonmotor symptoms (cognitive disturbance, pain, etc.), ALS is currently viewed more as a multisystem degeneration [3]. Moreover, behind the unique term “ALS” (the two beginning forms are those with spinal or bulbar onset), there is a spectrum of various phenotypes of the disease, leading to difficulty in making an early diagnosis [4]. Although the incidence of this fatal disease will probably increase in the coming years, mainly due to the ageing of the population [5], ALS remains rare: its incidence varies from 0.62/100,000 in Japan to 2.88/100,000 in the Faroe Islands; in France, the 2006 incidence was estimated to be 1.68/100,000 [6]. Despite many preclinical and clinical studies in the recent decades, the only effective treatment is riluzole, which modestly extends survival of ALS patients for 3 months after 18 months of treatment; additional treatments will likely be used in the future (RNA therapies, etc.), providing hope of a “polytherapy” approach with the hope to slow the evolution down and prolongs survival [4, 7]. The recent progress in gene therapy also highlight promising future antisense oligonucleotide and viral therapeutic strategies for treating ALS (particularly familial ALS) [8]. These new therapies will likely to be more effective if started in the earlier phase of the disease, which should then be diagnosed quickly. The most recent example of this need for a rapid diagnosis comes from the recently approved drug edaravone (Radicava—Mitsubishi Tanabe Pharma) which is only usable in the very early stages of the disease [9]. Many new clinical trials now include in their inclusion criteria a maximum delay for onset symptoms ranging from 18 to 24 months together with a preserved respiratory function. Earlier diagnosis also provides an opportunity for both patient and practitioner to anticipate complications and improve disease management, such as the use of noninvasive ventilation for respiratory impairment.

Despite this need for early diagnosis, ALS diagnostic delay (DiDe), corresponding to the period between the onset of symptoms and a positive diagnosis, has a surprisingly large range depending on the study, with a median delay of 12 months [10, 11], thus sometimes representing up to two-thirds of the total duration of the disease. The primary aim of our study was to better understand the origin of this delay in a cohort of patients living in southwest France (followed in the ALS Center of Bordeaux). We therefore determine the intermediate steps leading to the diagnosis (from the initial symptoms to the first consultation with a general practitioner—GP—to a consultation with specialist doctors or to complementary examinations) that may be shortened to decrease the delay in diagnosis of ALS. As GP is usually the first medical consultant and considering the low prevalence of the disease, many of these practitioners may have never faced a patient with ALS, specifically at the earlier phase (when the diagnosis may be difficult). The secondary aim of this study was then to describe a set of simple clinical “red flag” symptoms for nonneurologist practitioners (including GP) in order to quickly refer the patient to a specialist or a specialized center to confirm the diagnosis.

## 2. Materials and Methods

### 2.1. Study Population

We collected information regarding a cohort of patients seen and registered in the ALS Center of Bordeaux (Department of Neurology, Pellegrin Hospital, CHU Bordeaux). Inclusion criteria were broad: all the patients with possible, probable, or definite ALS according to the Awaji criteria [12] between 1 January 2013 and 31 December 2013 (in which the date of the first symptoms and diagnosis was recorded) were included. The only exclusion criterion was an obvious false-positive ALS diagnosis at the time of the study (respective to new data following the course of the disease). In total, 127 patients were registered as “new patients” in 2013, but 37 were excluded for various reasons (refusal of follow-up, not attending appointments, and premature death of the patient without a determined cause). Finally, 90 “new ALS patients” were included in our study.

### 2.2. Data Collection

We analyzed the data collected during consultations, diagnostic tests, clinical interventions, and hospitalizations of the patients. The time from the first symptoms to key milestones in the patients' journey to the ALS Center was studied. The milestones include GP consultations, specialist practitioner (SP: neurologist, pneumologist, ear-nose-throat or ENT, specialist, rheumatologist, etc.) consultations, referral to other health care professionals (orthophonist, physiotherapist, etc.), attendance at the ALS Center of Bordeaux, and diagnosis. For the first clinical symptoms/signs observed, patients were divided into “bulbar-onset,” “spinal-onset,” or “mixed-onset” ALS (when combination of both bulbar and spinal symptoms/signs was observed at the onset of the disease). We also collected all the spinal and/or bulbar symptoms/signs observed for each patient at various stages of the disease.

The primary endpoint of the study was the diagnostic delay (in months), defined as the period between the onset of the first symptom(s) and the final positive diagnosis, but many intermediate steps were also defined as follows: A: date of the first symptom(s) confirmed by a physician; B: date of the first complaint of the patient to the GP; C: date of the first consultation to a neurologist; D: date of the first electroneuromyography (ENMG); D^0^: date of the first suspicion of ALS; D^1^: date of the first ENMG with electrodiagnosis for ALS; E: date of the diagnosis announcement of ALS. Based on these intermediate steps, we calculated some delays (in months) used as secondary endpoints: delay AE (“global diagnostic delay”), delay AB (“delay of complaints”), delay BC (“delay before neurologist consultation”), delay CD^0^ (“delay to perform first ENMG”), delay AD^0^ (“delay to suspect ALS”), delay D^0^D^1^ (“delay for ALS suspicion”), delay D^1^D^2^ (“delay of the progression of the electrophysiological signs”), delay D^2^E (“delay to eliminate differential diagnosis”), and delay D^0^E (“delay for diagnosis confirmation”). For the calculation of each delay, the medians and averages were given.

### 2.3. Review of Literature

To compare our results with previous studies, we searched (via *PubMed* and *Google Scholar*) for all studies with data on the diagnostic delay in ALS between 1996 and 2017. When possible, we collected all the median and average values regarding diagnosis delay and every intermediate steps.

### 2.4. Statistical Analysis

All statistical analyses were performed using *Microsoft*® *Excel* 2013 for Windows®. The averages and medians were calculated using classical statistical formulas. The variance and standard deviations were given with the standard formula (*n* − 1), as it was a sample resulting from a population in which variables could fluctuate. The confidence interval was proposed by assuming compliance with Student's law. Student's *t*-test was carried out to compare the averages, with unilateral distributions on two samples of different variances; in the case of groups with fewer than 25 patients, it was assumed that the collected data followed a normal distribution. The *p* value corresponded to the validity of the assumption with an alpha risk (5%) to be mistaken: significance was considered at *p* < 0.05.

## 3. Results

### 3.1. Demographic Features at Diagnosis of ALS in Our Cohort

Among the 90 patients included in the study, 50 were male, 40 were female (sex ratio M/F: 1.25). The mean age at diagnosis was 67 for males and 67.5 for females. The mean weight at diagnosis was 72.1 kg for males and 59.9 kg for females. The mean BMI (body mass index) at diagnosis was 24.7 for males and 24.2 for females. The mean MMS (mini mental state) score at diagnosis was 26.3/30 (*n* = 40), and the mean FAB (frontal assessment battery) at diagnosis was 14.5/18 (*n* = 38).

“Spinal-onset ALS” was observed in 65.6% of the patients (*n* = 90): 76% of males and 52.5% of females. “Bulbar-onset ALS” was observed in 24.4% of the patients (*n* = 90): 12% of males and 40% of females. “Mixed-onset ALS” was observed in 10% of the patients: 12% of males and in 7.5% of females.

Based on the French demographic statistics of the INSEE (*Institut National de la Statistique et des Études économiques*) [13], the incidence of ALS in our area was estimated to be 2.17/100,000.

### 3.2. Diagnostic Delay of ALS and Intermediate Delays in Our Patients

**Figure 1:**
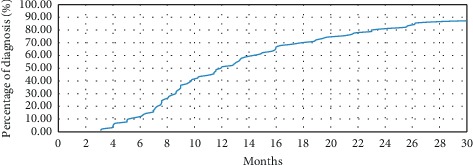
Rate of cumulative diagnoses of amyotrophic lateral sclerosis in function of time.

**Figure 2:**
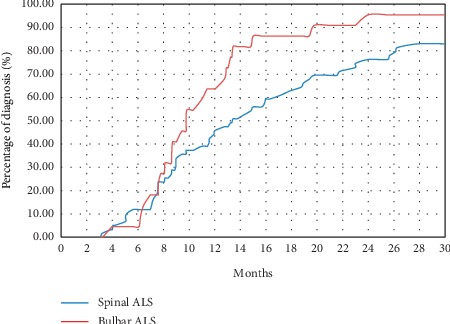
Rate of cumulative diagnoses accumulated according to time and clinical phenotype (bulbar or spinal form) at the onset of amyotrophic lateral sclerosis (ALS).

The median “diagnostic delay” (DiDe: AE delay) of ALS was 12 [3.1–89] months, and the average DiDe of ALS was 17 [13.1–21.3] months: the diagnosis of ALS was made within the first year of the appearance of symptoms in half of the patients (Figure 1). For bulbar ALS (*n* = 22), the median DiDe was 10 [4–43.8] months and the average DiDe was 12.6 [8.3–17.8] months. For spinal ALS (*n* = 59), the median DiDe was 13.4 [3.2–89] months and the average DiDe was 19.3 [14.1–25] months. For mixed ALS (*n* = 9), the median DiDe was 10.3 [3.1–30.9] months and the average DiDe was 12.7 months (Figure 2).

All the patients were first seen by GP. After this first consultation, if GP made no diagnosis and sent the patient home without additional medical examination (*n* = 29), the final median DiDe was 14.8 months and the average DiDe was 17.6 months. When GP immediately sent the patient to a neurologist (*n* = 19), the final median DiDe was 12.8 months and the average DiDe was 19.3 months. If the GP proposed an additional medical examination (*n* = 17), the final median DiDe was 9.8 months and the average DiDe was 16.3 months. Finally, if GP sent the patient to another nonneurologist specialist (*n* = 15), the final median DiDe was 8.7 months and the average DiDe was 9.4 months.

The median “delay of complaints” (AB) was 1.1 months, and the average AB was 2.7 [0.9–5.8] months, which represented 15.9% of the total DiDe. For bulbar ALS, the median AB was 1.2 months and the average AB was 2.2 months. For spinal ALS, the median AB was 1 month and the average AB was 3 months. When the GP sent the patient back without additional medical examinations, the symptoms were more recent (average: 1 month). The average delay for a neurological consultation was then 10 months. However, when GP directly referred the patient to a neurologist, the symptoms were older (average: 4 months) and the average delay to see a neurologist was 5 months (*p*=0.01).

**Figure 3:**
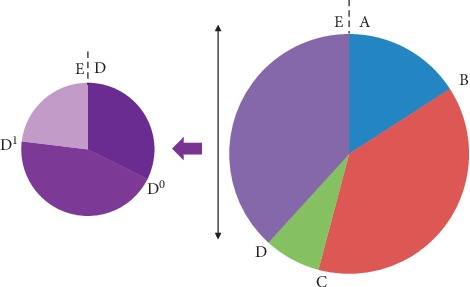
Presentation of the various stages leading to the diagnosis of amyotrophic lateral sclerosis (ALS). A: date of the first symptoms confirmed by a physician; B: date of the first complaint of the patient to GP; C: date of the first consultation to a neurologist; D: date of the first electroneuromyography (ENMG); D^0^: date of the first suspicion of ALS; D^1^: date of the first ENMG giving clear argues for ALS; E: date of the diagnostic announcement of ALS.

After the first visit to GP, the median “delay to visit the neurologist” (BC) was 4 months and the average BC was 6.5 [3.8–9.7] months (Figure 3), representing 38.4% of the DiDe. From the first visit to the neurologist, the median “delay to perform ENMG” (CD) was 0 months and the average CD was 1.3 months [0.4–2.6], representing 7.5% of the total DiDe. Half of the patients received this ENMG the same day as the first consultation, and ¾ of them received it during the month following this consultation. ENMG was performed in most of the patients before a real ALS suspicion. There was no BC difference between bulbar and spinal-onset ALS (*p*=0.45). From the first ENMG, the median “delay to suspect ALS” (DD^0^) was 0 months, and the average DD^0^ was 2.1 [0–4.7] months. In most cases (64%), ALS was suspected after the first ENMG; in other cases, ALS was suspected before the first ENMG. When ALS was suspected, the median “delay for diagnosis confirmation” (DD^1^) was 0.2 months and the average DD^1^ was 2.9 [1.2–4.9] months. In half of the patients (55.6%), only one ENMG was needed to confirm ALS.

Finally, for all the patients, the average delay before the first consultation to the ALS Center was 17.5 months (median delay was 12.2 months).

### 3.3. Diagnosis Delay of ALS and Survival

**Figure 4:**
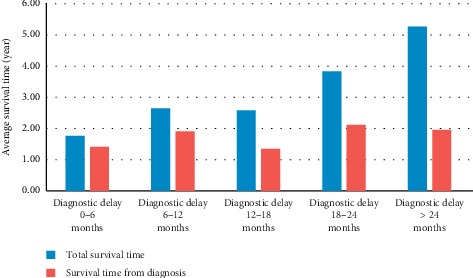
Analysis of life expectancies of deceased patients in function of the diagnostic delay.

Among the 90 patients, 69 (76.7%) were dead when the study was closed. The median survival time (since the first symptoms) of all 90 patients was 3.1 years. For deceased patients, the median survival time (since the first symptoms) was 2.95 years, which can be divided into the average DiDe (1.18 years) and the average time of survival after diagnosis (1.77 years). Thus, for deceased patients, DiDe represented 40% of the total survival since the first symptoms (Figure 4). For living patients, the first symptoms began 3.87 to 10.34 years earlier.

After a review of the literature, there were a total of 27 studies (including our study) with data regarding diagnostic delay in ALS. The detailed results are summarized in Table 1.

### 3.4. Clinical Symptoms Observed in Our Patients

The main symptoms observed in our patients are summarized in Table 2. Whether it was the stage of the diseases (at the time of the first symptoms of the first examination of GP or by the neurologist), the three most common symptoms were, in order of occurrence: motor weakness of the lower limbs (MWLL), motor weakness of the upper limbs (MWUL), and speech disability (SpD). At the time of diagnosis, if motor weakness of the limbs (MWL) remained the most frequent (predominantly in the upper limbs), fasciculations (in lower or upper limbs) were a little more present than SpD. Breathing disorders, swallowing disability (SwD), and upper limb muscle atrophy were also present in more than half of the patients. The general sensitivity of MWL, SpD, breathing disability, and SwD was greater than 85%.

With regard to onset delay, the first three symptoms that appeared before diagnosis were (in chronological order): MWLL, SpD, and then MWUL. These symptoms appeared more than 3 months before diagnosis (median delay). Regardless of the stage of the disease, MWL and SpD appeared to be the most frequent, with a very good general sensitivity and interesting onset delay in relation to diagnosis. A “clinical triad” could thus be established, combining exhaustively MWUL, MWLL, and SpD (thus, the “clinical duo” SpD + MWL, whether upper or lower, emerged). The presence of this “clinical triad” was verified in 2.2% of the cases at the beginning of symptoms, 3.3% at the time of the first examination by GP, 17.8% at the first examination by the neurologist, and 36.7% at the time of the diagnostic announcement. Finally, 81.1% of ALS patients had this triad at the end of their disease progression. Concerning the other “clinical duo” SwD + MWL, 6.7% of patients expressed it at the beginning of the symptoms, 8.9% at the time of the first examination by GP, 27.8% at the time of the first examination by the neurologist; 48.9% of the patients were finally affected by this duo at the time of diagnosis: its general sensitivity was 88.9% and its median occurrence was 0.1 months before the diagnostic announcement. Surprisingly, very little or no description of the reflexes was found in the referral description from the GP. This probably suggests that most GP do not consider reflexes as an important sign although it is a cardinal sign for ALS diagnosis.

## 4. Discussion

In many ways, our cohort appears similar to those of other epidemiological studies of greater size. Thus, we found the sex ratio, distribution of symptoms (bulbar/spinal) according to sex, and mean age of occurrence of the disease similar to the results observed in a recent Italian study [1]. The estimated incidence of ALS in our sample also appears close to the expected incidence of ALS in the French population [14, 15]. Finally, our population, although comprising a small number of patients (90), appears representative of the general population of ALS patients.

As observed in other studies [16], most patients receive a diagnosis of ALS in the year following the appearance of the first symptoms; however, longer DiDe can be observed. As indicated in Table 1 for most of the studies, the median DiDe is estimated to 12.1 months and the average DiDe is estimated to 14.2 months. However, if our median DiDe is similar (12 months), our average DiDe appears to be much longer than it should be (17 months). This finding can be explained by our choice regarding the date of diagnosis of ALS and even the date of the occurrence of the first symptoms of the patient. Indeed, many studies considered the diagnosis of ALS made as soon as it was suspected, which can be questionable; in our case, we considered that the diagnosis was made if and only if the diagnosis had been announced to the patient, which is, in our view, closer to real life and, more importantly, better reflects the waiting period for the patient before a clear diagnosis and start of specialized medical care. In some studies, the diagnosis corresponds to the first consultation to the neurologist [10], which might not be exact in all circumstances, as observed in our study. Other authors considered all “motor neuron disorders” [17], thus including pathologies other than ALS. In addition, even if precise data can be reliably obtained from the patient's memory concerning his/her first symptoms (which still has a risk of memory bias) [18–21], we chose to consider only the data clearly written in medical documents. “Diagnostic delay cutoffs,” was often arbitrarily given in some studies; for example, patients were sometimes excluded if the time to diagnose ALS was greater than 18 months [18], 30 months [22], or 36 months [23]. Similarly, Househam and Swash did not include patients when duration of some intermediate stages (a disproportionately long time from the first medical contact with seeing a neurologist) was greater than 6 months [20]. This lack of standardization in the data collection methodology likely explains the great variability between the various studies. According to the methodology used, DiDe could easily double for the same patient.

Interestingly, DiDe depends on the sequence of successive medical choices, starting with the first doctor, which is often GP (especially in France where all patients have to be addressed to GP before any consultation with SP). However, our study found no significant influence on DiDe whether GP referred a patient directly to a neurologist or sent the patient back home without further examination (*p*=0.36). The only case with a significantly shorter DiDe was when GP referred the patient to a nonneurologist specialist, which is surprising for a neurological disease. Nevertheless, sending a patient to another specialist means that more specific signs have appeared (respiratory failure and swallowing difficulty), which more easily allow a quick final diagnosis. Moreover, as shown in other studies, we found a shorter DiDe in bulbar forms, for which GP is likely to refer the ENT physician. Therefore, the initial choice of GP has no major influence on DiDe. On the other hand, further reassessment and the number of physicians involved in the diagnosis process seem to be important factors in lengthening DiDe. It is therefore probably more important to further analyze intermediate delays to capture the key elements susceptible to shorten the DiDe.

The “delay of complaints” (AB), representing 15.9% of the total DiDe, is probably the first delay that could be reduced; such a patient-dependent delay can be explained by the patient's disregard of early signs. However, as the AB delay is shorter in our study than in other studies (Table 1), it is probably not the primary reason. A key step to be shortened is likely the “delay for consultation to the neurologist” (BC), representing 38.2% of the total DiDe. We have found that a third of the patients were sent back home (without further physical examination or medical test) after the first GP consultation. However, when patients have additional examinations (scanner, MRI, etc.), the delay to consult a neurologist was halved. This result fits well the “all or nothing law” described in a French study in 2006, which is likely dependent on the first symptoms [10]. At this stage, bulbar involvement will likely prompt a nonneurologist physician to ask for complementary investigations rather than just fasciculations in the limbs. We found that only one-fifth of the patients were referred to a neurologist after the first GP examination (with an average delay of only 1 month to obtain such a consultation if decided by GP). In our study, it seems clear that GPs probably wait too long and for overly specific symptoms before sending the patient to a neurologist. As soon as the neurologist sees the patient, he/she usually performs ENMG very quickly (CD^0^). This delay represents only 7.5% of the total DiDe, indicating that there is usually rapid access to the electrodiagnosis. However, if the neurologists usually quickly perform the first ENMG, they also sometimes need successive ENMGs and/or clinical examinations to certify the diagnosis according to the current criteria (especially in the forms without bulbar or respiratory symptoms), explaining the longer delay sometimes obtained by the neurologists. Efforts are therefore probably necessary to ensure that GPs have sufficient knowledge about ALS (and its consequences for care and treatment) to sensitize them as much as possible, but it is also important for the neurologists not to delay referring the patient to an ALS clinic if they already have reasonable doubts about the diagnosis after their first ENMG. Along the same line, a probable lack of knowledge leads nonneurologist physicians (GP or not), also lacking clear and simple guidelines, to quickly identify symptoms that should clearly be considered as “red flags” for a severe neurological disorder and more specifically ALS.

**Figure 5:**
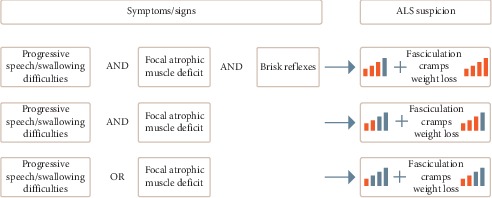
Proposal for a decision-making diagram to quickly evocate the diagnosis of amyotrophic lateral sclerosis according to the observed clinical symptoms/signs.

To help to identify those “red flags,” we propose a minimal set of symptoms that should be searched and identified for each patient with any neurological disorders (Figure 5). Two situations can be differentiated: (a) a patient with progressive SpD should be clearly considered for bulbar-onset ALS; additional symptoms such as tongue atrophy, and, of course, SwD, sometimes only be silent aspiration (detectable only by coughing after drinking or eating); a loss of weight should be suspicious too (in part due to muscle atrophy); (b) any focal asymmetric motor deficit, with no sensitivity loss but with muscular atrophy, should be considered as a possible sign of onset of ALS. In both situations, pyramidal signs (such as brisk reflexes) should be systematically searched, even if their absence in bulbar onset is common; but their presence, together with any muscular atrophic region (tongue and dorsal interosseous of the hand, specifically if asymmetric) should be considered as highly suspicious of ALS. Finally, fasciculation and cramps, often early noticed by the patients themselves, when persistent and diffuse, should also be considered as pathological. Those signs should promptly direct to a neurologist for quick clinical and ENMG examination.

## 5. Conclusions

The diagnosis delay of ALS is influenced by many factors, and is certainly not incompressible. More than half of this time is lost before the first visit to the neurologist, with a particularly important role of GP. GP's first choices will determine the remainder of the diagnosis process until the diagnosis of ALS. A shortening of the diagnostic delay could therefore be gained at each intermediate stage of the patient's care path, particularly during the initial part. It would therefore seem necessary to better inform nonneurologist physicians about this disease (and its consequences); so we propose a table with a set of easily recognizable symptoms to quickly assess the possibility of such a fatal disease: this tool may help nonneurologist practitioners to diagnose the classical form of the disease, probably not for the less common and atypical forms (such as flail arm syndrome).

## Figures and Tables

**Table 1:** Diagnostic delay and intermediate delays of amyotrophic lateral sclerosis in the medical literature.

Reference	Country	Number of patients	Diagnostic delay		Intermediate delays	
			Median (months)	Average (months)	Median (months)	Average (months)
Belsh and Schiffman [19]	USA	64	—	12.3	—	A-B (5.9)
Brooks [11]	USA	34	—	21.9	—	C-E (8.7)
Chiò [22]	International	201	14	17–21	A-B (2); B–C (6); C-E (4)	A-B (5.6); B–C (6); C-E (7)
Househam and Swash [20]	UK	57	—	16.2	A-B (3); B–C (5); C-E (2)	A-B (5.2); B–C (7.2); C-E (3.8)
Traynor et al. [23]	Ireland	388	8	—	—	—
Iwasaki et al. [24]	Japan	117	—	11.7–12.7	—	A-B (5.7); A-C (9.7)
Chiò et al. [25]	Italy	221	—	11.1	—	—
Zoccolella et al. [18]	Italy	130	9.3	—	—	—
Czaplinski et al. [26]	USA	1041	13–14.2	—	—	—
Torny et al. [10]	France	77	7	11.7	A-B (2); B–C (3)	A-B (4.7); B–C (6.9)
Donaghy et al. [17]	Northern Ireland	73	15.6	—	A-B (3.1); B–C (5.2); C-E (1.9)	—
Chiò et al. [27]	Italy	1260	—	10.4	—	—
Kraemer et al. [28]	Germany	100	—	13.7	—	—
Mitchell et al. [16]	England	640	11.4	—	—	—
Turner et al. [29]	Ireland	49	9–15	10–22	—	—
Logroscino et al. [6]	Europe	1028	8.7	12.2	—	—
Cellura et al. [30]	Italy	260	11	—	A-B (3); B–C (3); C-E (5)	—
Williams et al. [31]	USA	272	27	—	—	—
Nzwalo et al. [32]	Portugal	101	9.5	10.1	A-B (2); A-C (6)	—
Paganoni et al. [33]	USA	304	11.5	—	A-B (4); B-D^0^ (3) – D^0^-E (1)	—
Scialo et al. [21]	Italy	298	10	14.3	—	—
Knibb et al. [34]	England	575	11.1	—	—	—
Stevic et al. [35]	Serbia	325	—	19.2	—	—
Yates and Rafiq [36]	Europe	512	—	9.5	—	—
Calvo et al. [1]	Italy	2648	14.2–15	—	—	—
Galvin et al. [37]	Ireland	155	11	15.1	A-B (3)	A-B (5.2); B–C (6.1); C-E (3.8)
Our study	France	90	12	17	A-B (1.1); B–C (4); A-D^0^ (0); C-D^0^ (0); D^0^-E (0.2)	A-B (2.7); B–C (6.5); A-D^0^ (2.1); C-D^0^ (1.3); D^0^-E (2.9)
Average		**408.1**	**12.1**	**14.2**	**A-B (2.6); B–C (4.4); C-E (3.2)**	**A-B (5); B–C (6.5); C-E (5.8)**
Range		**34–2648**	**7–27**	**9.5–21**	**A-B (1.1-4); B–C (3-6); C-E (1.9-5)**	**A-B (2.7-5.9); B–C (6-7.2); C-E (3.8-8.7)**

A-B = delay of complaint; A-C = delay for a neurologist consultation since the first symptom of the patient; A-D^0^ = delay of the first suspicion of ALS since the first symptom of the patient; B–C = delay for a neurologist consultation since the first complaint of the patient; B-D^0^ = delay of the first suspicion of ALS since the first complaint of the patient; C-D^0^ = delay to perform electroneuromyography; C-E = delay for the neurologist to make the diagnosis of ALS; D^0^-E = delay for diagnosis confirmation.

**Table 2:** Occurrence of bulbar and spinal signs in our patients.

		At first feeling by the patient (%)	At first examination by GP (%)	At first examination by the neurologist (%)	At diagnosis (%)	General prevalence (%)
Bulbar signs	Speech disability	32.22	33.33	47.78	61.11	92.22
	Breathing disability	6.67	10	22.22	52.22	92.22
	Swallowing disability	13.33	13.33	37.78	51.11	86.67
	Excessive saliva	5.56	6.67	11.11	20	42.22
	Tongue fasciculations	1.11	3.33	16.67	41.11	61.11
	Tongue atrophy	0	0	5.56	16.67	40
	PCL	1.11	1.11	2.22	7.78	18.89

Spinal signs	Hoffmann sign	0	0	8.89	42.22	56.67
	LL motor weakness	42.22	47.78	57.78	70	91.11
	UL motor weakness	31.11	35.56	52.22	72.22	93.33
	LL fasciculations	7.78	11.11	30	67.78	74.44
	UL fasciculations	6.67	8.89	36.67	65.56	71.11
	Body fasciculations	3.33	4.44	11.11	22.22	27.78
	LL muscle atrophy	0	1.11	10	27.78	37.78
	UL muscle atrophy	6.67	8.89	30	57.78	67.78
	LL cramps/pain/spasticity	21.11	24.44	33.33	47.78	66.67
	UL cramps/pain/spasticity	12.22	18.89	27.78	42.22	66.67
	Body cramps/pain/spasticity	1.11	1.11	2.22	5.56	14.44

LL: lower limbs; PCL: pathological crying and laughing; UL: upper limbs.

## Data Availability

The data used to support the findings of this study are available from the corresponding author upon request.
